# Dihydroquercetin Attenuates Silica‐Induced Pulmonary Fibrosis by Modulating the Gut Microbiota and the Serum Metabolites in Mice

**DOI:** 10.1002/fsn3.71389

**Published:** 2026-01-07

**Authors:** Zunqiong Ke, Lishu Lin, Yunhong Long, Wenhui Zhang, Jianmin Guo, Leyong Yuan

**Affiliations:** ^1^ Department of Clinical Laboratory The Second People's Hospital of Futian District Shenzhen Shenzhen China; ^2^ Department of Pharmacy Peking University Shenzhen Hospital Shenzhen China; ^3^ Department of Health Management, The Eighth Affiliated Hospital Sun Yat‐Sen University Shenzhen China

**Keywords:** correlation analysis, dihydroquercetin, gut microbiota, pulmonary fibrosis, serum metabolites

## Abstract

Dihydroquercetin (DHQ), a crucial dihydroflavone found in nature, demonstrates notable antioxidant, anti‐inflammatory, and antifibrotic effects. Nevertheless, the impact on the gut microbiome and metabolites associated with silicosis remains unclear. Hence, the objective of this research was to examine how DHQ impacts silicosis and the associated mechanisms through analyzing gut microbiota with 16S rRNA sequencing and conducting serum metabolomic analysis. The findings of our study showed that administering DHQ significantly attenuated the level of inflammation and fibrosis in the lung tissues of C57BL/6 mice exposed to silica. Furthermore, DHQ clearly raised the amount of Muribaculaceae, while diminishing the amount of *Lactobacillus*. DHQ treatment significantly decreased the sphingomyelin, arachidonic acid and its metabolites. Significantly, the correlation analysis showed that the influence of DHQ on the arachidonic acid metabolism, steroid hormone biosynthesis, and sphingolipid signaling pathways were linked to changes in the levels of *Muribaculaceae* and *Lactobacillus* in the gut microflora. In summary, our research demonstrated that DHQ can attenuated inflammation and lung fibrosis caused by silica exposure in the C57BL/6 mice, potentially by modulating the gut microbiota and serum metabolites.

## Graphical abstract

(1) DHQ can attenuate silica‐induced inflammation and pulmonary fibrosis in the C57BL/6 mice. (2) The beneficial effects of DHQ were primarily attributed to the alteration of gut microbiota (particularly Muribaculaceae and Lactobacillus) mediated by serum metabolites.

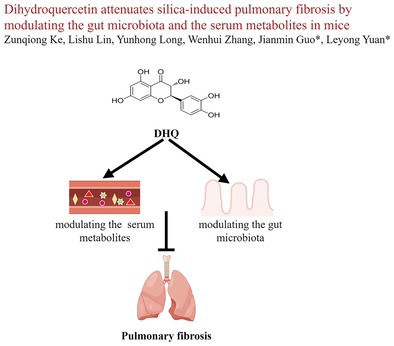

## 1 Introduction

Pulmonary fibrosis (PF) represents the final stage of a range of diverse interstitial lung diseases. Pulmonary fibrosis is characterized by inflammation, damage to alveolar epithelial cells, extensive lung fibroblast proliferation, and irregularities in lung tissue repair and remodeling, leading to an average lifespan of merely 3 to 5 years (Fang et al. 2025; Sun et al. 2024). The reported incidence of pulmonary fibrosis (PF) varies significantly, ranging from 0.9 to 9.3 cases per 100,000 individuals in Europe and North America, and from 3.5 to 13.0 cases per 100,000 individuals in Asia and South America (Hu et al. 2024). Silicosis, a common and severe occupational lung disease, is a typical form of pulmonary fibrosis caused by the inhalation of large amounts of crystalline silica dust (Churg and Muller 2024; Leung et al. 2012). Despite the limited understanding of the underlying mechanisms of pulmonary fibrosis and silicosis, the prevailing paradigm of disease pathogenesis incorporates specific cellular processes and molecular pathways. The initial injury to the alveolar epithelium initiates a cascade of events, including inflammation and subsequent alveolar epithelial damage, which are critical for the disease's onset (Borok et al. 2020; Watanabe et al. 2021). Following this initial injury, lung fibroblasts are activated and differentiate into myofibroblasts in response to the release of cytokines and growth factors from the compromised lung epithelium, alongside the activation of pro‐fibrotic signaling pathways. This sequence of events ultimately results in the deposition of extracellular matrix (ECM) components and the formation of progressive fibrotic scars within the lung parenchyma, leading to impaired lung function (Borchers et al. 2011; Tzouvelekis et al. 2019). Consequently, therapeutic strategies primarily focus on inhibiting fibrotic factors, reducing lung inflammation, suppressing the proliferation and activation of interstitial cells, and modulating the synthesis and degradation of ECM components, thereby reducing lung tissue injury and formation of pulmonary fibrosis (Liu et al. 2016; Pang et al. 2019). Pirfenidone, poly‐2‐vinylpyridine‐N‐oxide (PVNO), nintedanib, and tetrandrine are among the drugs currently prescribed for pulmonary fibrosis or silicosis, but their therapeutic effects are limited (Ernst et al. 2002; Pang et al. 2019). Therefore, developing better treatment strategies is crucial.

Recent research indicates that dysbiosis of the gut microbiota is linked to various pulmonary conditions, including lung cancer, asthma, chronic obstructive pulmonary disease (COPD), and acute lung injury (Barcik et al. 2020; Ma et al. 2022; Qu et al. 2022; Tan et al. 2020; Zhao et al. 2021). The gut microbiota influences a wide array of physiological functions in the host, such as metabolism, immune defense, and cognitive neurodevelopment. Notably, the gut microbiota may affect metabolite levels, which could serve as direct indicators of the current metabolic state of organs or cells (Tremaroli and Backhed 2012). The use of 16S rRNA sequencing alongside metabolomics can help uncover the mechanisms of Chinese herbal formulas by studying the interaction between gut microbiota and host metabolism. Piao et al. reported the anti‐aging properties of Fu‐Fang‐Zhen‐Zhu‐Tiao‐Zhi capsules through increasing Short‐chain fatty acids producing bacteria, reducing the presence of bacteria that produce hydrogen sulfide and augmenting glucose and lipid metabolism (Shenghua et al. 2020). The study discovered that Kang‐Shuai‐Lao‐Pian impacted the levels of *Christensenellaceae_R‐7_group*, *Intestinimonas*, *Oscillibacter*, and *Lachnoclostridium_UCG‐010*. Meanwhile, *Aliihoeflea* affected the metabolism of lysine, dipeptides, fatty acids, and purines in obese mice (Gong et al. 2020).

Dihydroquercetin (DHQ) is a common flavonoid, which is found in the bark of yew, larch, and cedrus brevifolia trees. Recent research has demonstrated the diverse pharmacological benefits of DHQ, including anti‐inflammatory, anti‐cancer, antioxidant, anti‐proliferative, and anti‐radiation effects (Y. Liu et al. 2023). Impellizzeri et al. reported that DHQ has shown an ability to attenuate bleomycin‐induced lung fibrosis (Impellizzeri et al. 2015). Yuan et al. demonstrated that DHQ markedly attenuates silica‐induced lung inflammation and fibrosis in mice (Yuan et al. 2022). Nevertheless, it remains uncertain how DHQ provides protection against silica‐induced pulmonary fibrosis. The silicosis model mice and DHQ treatment regimen were established to further understand these mechanisms. The 16S rRNA sequencing was utilized to examine the alterations in gut microbiota following DHQ treatment. Furthermore, untargeted metabolomics was conducted to explore the metabolic effects of DHQ on silicosis. Comprehending the mechanism by which DHQ improves silicosis can offer clinicians an additional effective treatment option and contribute to uncovering the connection among Chinese herbal remedies, gut bacteria, and blood metabolites.

## 2 Materials and Methods

### 2.1 Reagents and Chemicals

Aladdin Biochemical Technology Co. Ltd. in Shanghai, China provided DHQ (Cat#480‐18‐2) with a purity exceeding 98%. Sigma‐Aldrich provided the SiO_2_ (Cat#S5631) particles (around 80% diameter 1‐5 μm), which were filtered through sedimentation following Stokes' law, underwent acidic hydrolysis, and were baked overnight at 200°C for 16 h. Interleukin‐1β (IL‐1β) (CSB‐E08054m), tumor necrosis factor‐α (TNF‐α) (CSB‐E04741m), and transforming growth factor‐β (TGF‐β) (CSB‐E04726m) were acquired from Cusabio Biotechnology in Wuhan, China. Cell Signaling Technology Inc. (Danvers, MA, USA) provided antibodies for alpha‐smooth muscle actin (α‐SMA) (#19245), Fibronectin (#26836), Collagen I (#72026), and GAPDH (#5174). Proteintech Biotechnology (Wuhan, China) provided a second antibody.

### 2.2 Animals and Procedures

The eight‐week‐old male C57BL/6 mice were obtained from Cloud‐Clone Corporation in Wuhan, China. A study using a mouse model for silica‐induced pulmonary fibrosis was executed. In short, mice in each group were anesthetized by intraperitoneal injection of 3.5% chloral hydrate solution (0.1 mL/10 g) and surgery was performed to expose their tracheae. Furthermore, a suspension of SiO_2_ (20 mg in 50 μL of saline) was administered to the mice and the control group received the same volume of 0.9% sterile saline. After acclimation for 1 week, mice were randomly divided into three groups (*n* = 6 per group): Control group, SiO_2_ group, DHQ group (DHQ, 50 mg/kg). A week after receiving SiO_2_, mice were given DHQ orally at a dose of 50 mg/kg daily for 14 days, while the control and SiO_2_ groups were given the same amount of 0.9% sterilized saline. An intraperitoneal injection of chloral hydrate was used to euthanize the mice on day 21. Samples were collected from each group for subsequent analysis. After removing and weighing lung tissues, the lung weight to body weight (LW/BW) ratio was calculated as the pulmonary index. The lung samples from the lower left lobe were preserved in 10% formalin for histopathological examination, while the rest of the samples were frozen at −80°C. All experimental procedures were executed according to the protocols approved by the Animal Ethics Committee, The Second People's Hospital of Futian District Shenzhen (ethic No. D202408‐9).

### 2.3 Gathering of Tissue Samples and Examination of Lung Histopathology

Lung tissues were quickly collected after sacrifice and treated for 24 h with 4% paraformaldehyde. Tissue samples were dehydrated using alcohol gradients and then encased in paraffin after being fixed. Next, the specimens were sliced into 5 μm segments and underwent staining with hematoxylin and eosin (H&E) or an enhanced version of masson trichrome. The lung tissue samples were examined and photographed using a light microscope.

### 2.4 Histological Scoring Evaluation, Inflammation Cytokines Detection, and Fibrosis Markers Detection

The approach detailed in our prior study was utilized to assess the inflammation and fibrosis scores, inflammation cytokines examination (such as IL‐1β, TNF‐α, and TGF‐β), and fibrosis markers (including α‐SMA, collagen I, and fibronectin) examination (Yuan et al. 2022).

### 2.5 Fecal 16S rRNA Sequencing Analysis

After the DHQ treatment, fecal samples were collected from the control, SiO_2_, and DHQ groups at the same time in a sterile environment within a laminar flow hood. Genomic DNA from the microbial community was obtained from samples by following the manufacturer's instructions using the PF Mag‐Bind Stool DNA Kit from Omega Bio‐tek in Georgia, U.S. The concentration and purity of the DNA sample were assessed with a NanoDrop 2000 UV–vis spectrophotometer from Thermo Scientific in Wilmington, USA, after being analyzed on a 1% agarose gel. For the bacterial community, universal bacterial primers were used to amplify the bacterial 16S rRNA genes (forward: 5′‐AGRGTTYGATYMTGGCTCAG‐3′) and (reverse: 5′‐RGYTACCTTGTTACGACTT‐3′). To differentiate each sample, primers were tagged with PacBio barcode sequences. A total volume of 20 μL was used for the amplification reactions, containing 5 × FastPfu buffer solution (4 μL), 5 μM each primer (0.8 μL), 2.5 mM dNTPs (2 μL), template DNA (10 ng), and FastPfu Polymerase (0.4 μL). Afterwards, the PCR samples were measured through 2% agarose gel electrophoresis and then cleaned with AMPure PB beads from Pacifc Biosciences in CA, USA. After combining the refined items in equal amounts, a DNA library was created with the SMRTbell prep kit 3.0 from Pacifc Biosciences, located in CA, USA, following the guidelines provided by PacBio. Majorbio Bio‐Pharm Technology Co. Ltd. (Shanghai, China) sequenced the clean SMRTbell libraries using the Pacbio Sequel IIe System from Pacifc Biosciences in CA, USA.

Following the completion of sequencing, the raw data underwent quality filtering and merging to obtain effective reads. The optimized sequences were subsequently clustered into operational taxonomic units (OTUs) using UPARSE version 11, with a sequence similarity threshold of 97%. Based on the OTU abundance profile, α‐diversity and β‐diversity analyses were conducted using the Majorbio Cloud Platform (www.majorbio.com). Within‐sample α‐diversity was evaluated using the Sobs, Shannon, Ace, and Chao indices. The visualization of between‐sample β‐diversity was achieved through a PCoA plot based on the binary Jaccard distance. Statistical significance for α‐diversity was assessed using the Kruskal‐Wallis H test, while β‐diversity was evaluated using ANOSIM analysis. The analysis of the relative abundance of key microbiomes at the phylum and genus levels was conducted using the Kruskal‐Wallis *H* test.

### 2.6 Untargeted Metabolomics Study

After the DHQ treatment, the serum samples from different treatment groups were collected for metabolomics analysis. A 100 μL liquid sample was combined with a 400 μL solution (acetonitrile methanol = 1:1 (v:v)) in a 1.5 mL centrifuge tube, which included 0.02 mg/mL internal standard (L‐2‐chlorophenylalanine) for the purpose of extracting metabolites. The specimens were vortexed for 30 s and then sonicated at low temperature for 30 min (5°C, 40 KHz). The specimens were stored at a temperature of −20°C for half an hour in order to separate the proteins. Following this, the specimens underwent centrifugation for a duration of 15 min at a temperature of 4°C and a speed of 13,000 g. The liquid above the sediment was extracted and dried using nitrogen gas. After that, the specimen was re‐dissolved using a 100 μL mixture of acetonitrile and water in equal parts, then subjected to low‐temperature ultrasonication for 5 min at 5°C and 40 KHz. The resulting solution was then centrifuged at 13,000 g and 4°C for 10 min. The liquid above the sediment was moved to sample containers for analysis using LC–MS/MS. LC–MS/MS analysis was performed using an ACQUITY HSS T3 column on a Thermo UHPLC system coupled with Q Exactive.

The raw UHPLC–MS/MS data files were imported into Progenesis QI 2.3 (Nonlinear Dynamics, Waters, USA) for peak detection and alignment analysis. Following baseline correction and screening processes, metabolite peak intensities were normalized to the total spectral intensity. Multivariate statistical analyses, including principal component analysis (PCA) and orthogonal partial least‐squares discriminant analysis (OPLS‐DA), were subsequently employed to evaluate the overall differences between groups. Metabolite identification was conducted based on adducts, molecular ion peaks, and fragments, with reference to the Human Metabolome Database (HMDB, http://www.hmdb.ca/) and ChemSpider (http://www.chemspider.com/). Differential metabolites were identified using criteria of VIP > 1, *p* < 0.05, and fold change (FC) ≥ 1.5 or FC ≤ 0.5 among the three groups. The fold change was expressed as the binary logarithm of the average normalized peak area ratio. The primary biochemical metabolic pathways and signal transduction pathways of the differential metabolites were analyzed using the Kyoto Encyclopedia of Genes and Genomes (KEGG) database (http://www.genome.jp/kegg).

### 2.7 Correlation Analysis Between Physiological Data, Serum Metabolites, and Gut Microbiota

Spearman's correlation between the physiological data (such as pulmonary index, inflammation and fibrosis scores, cytokines, and fibrotic markers), the levels of serum metabolites, and gut microbiota components at the genus level was analyzed.

### 2.8 Statistical Analyses

GraphPad Prism version 8 was used to conduct statistical analyses, and data were shown as mean with standard deviation (SD). For multiple group comparisons, a one‐way ANOVA was conducted. Significance was determined for differences with a *p* value less than 0.05.

## 3 Results

### 3.1 Effects of DHQ on Pulmonary Fibrosis and Inflammatory Infiltration in Silicosis Model Mice

**FIGURE 1:**
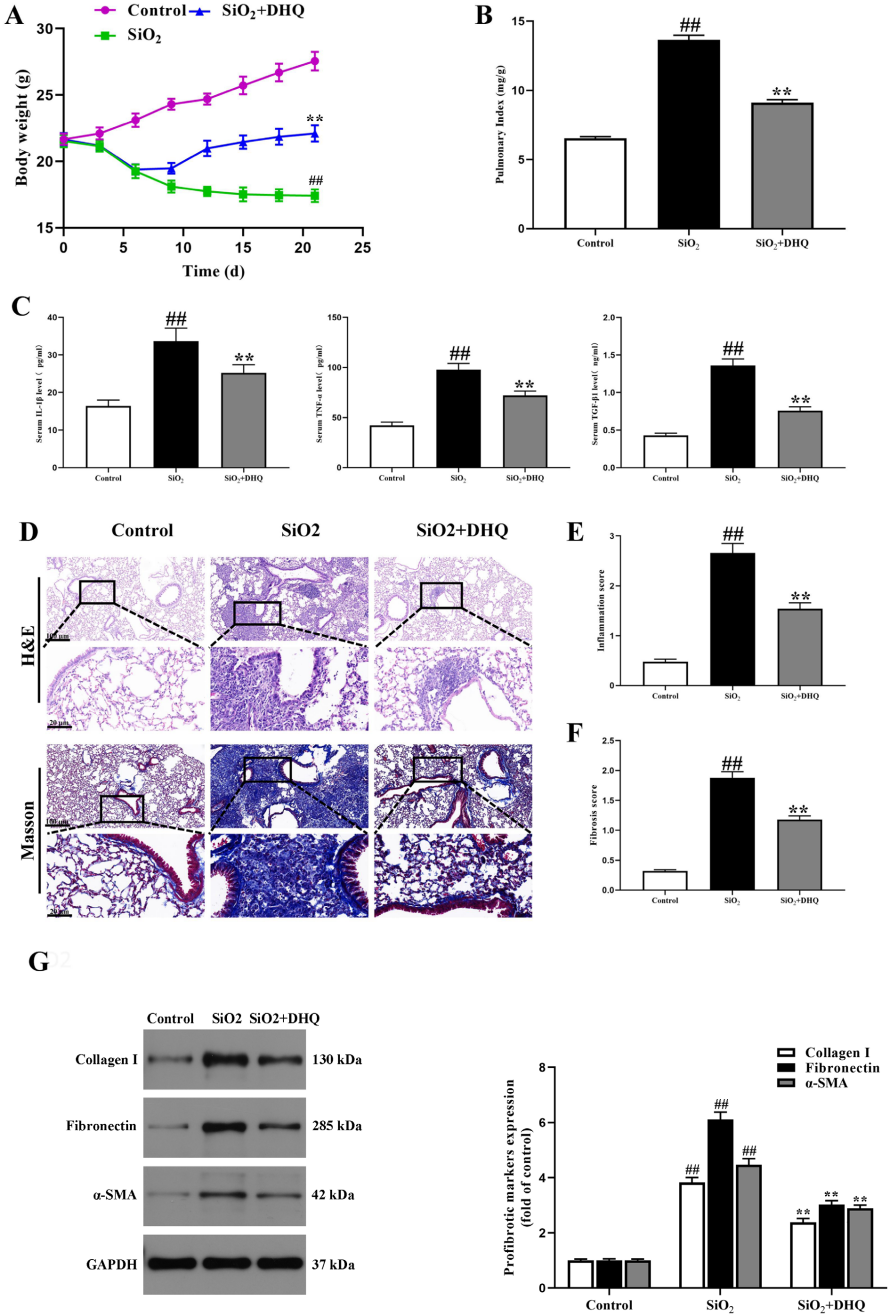
DHQ treatment attenuated silica‐induced pulmonary fibrosis in C57/BL6 mice. (A, B) DHQ treatment increased the body weight and decreased pulmonary index in silicosis model mice. The changes of body weight (C) The levels of pro‐inflammatory cytokines (IL‐1β, TNF‐α, and TGF‐β) in serum from different groups at day 21 were detected by ELISA assay. (D) Representative pictures (×200) of HE‐stained and Masson‐stained lung sections from mice on day 21 were shown. Bar = 100 μm. (E, F) The inflammation and fibrosis score numbers of 0–3, corresponding to the grades of –, +, ++, and +++, were evaluated by experienced pathologists in a blinded fashion. (G) Representative results of western blot for α‐SMA, collagen I and fibronectin in lung tissues and the quantification of results. Data are shown as mean ± SD. All experiments were repeated three times. #*p* < 0.05, ##*p* < 0.01 vs. the control group; *p < 0.05, **p < 0.01 vs. the SiO_2_ group.

Following a 2‐week DHQ treatment at a dosage of 50 mg/kg, there was a notable increase in body weight, along with a decrease in pulmonary index and serum levels of IL‐1β, TNF‐α, and TGF‐β in the DHQ group when compared to the SiO_2_ group (Figure 1A–C). When comparing the DHQ group to the SiO_2_ group on the day 21, histological examination and masson's staining revealed that DHQ improved alveolar walls, attenuated inflammation, and decreased collagen fiber deposition. Likewise, the DHQ groups showed a lower inflammation and fibrosis score compared with the SiO_2_ group (Figure 1D–F). Furthermore, DHQ treatment in mice led to a significant decrease in the levels of α‐SMA, fibronectin, and collagen I compared to the SiO_2_ group, as shown in Figure 1G. Overall, these results provide additional evidence that DHQ has the potential to significantly reduce inflammation and fibrosis caused by silica in silicosis model mice.

### 3.2 Modulatory Effects of DHQ on Gut Microbiota in Silicosis Model Mice

**FIGURE 2:**
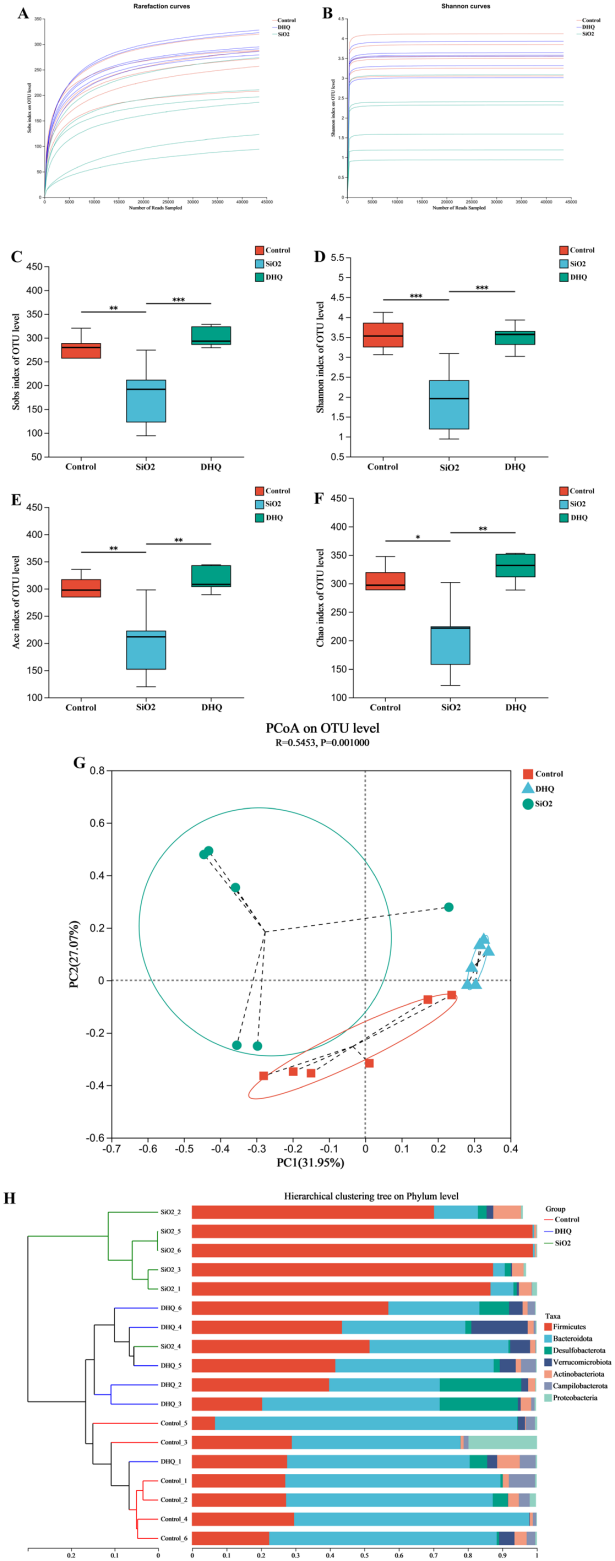
Overall structural changes in microbiota composition (A, B) Rarefaction curve calculated indexes of Sobs and Shannon on OTU level. (C–F) Alpha diversity estimators calculated indexes of Sobs, Shannon, Ace, and Chao on OTU level. (G) Principal component analysis (PCA) of all samples by Bray‐Curtis distance. (H) Hierarchical clustering analysis of all samples on the phylum level. Data are presented as mean ± SD, *p < 0.05, **p < 0.01.

The 16S rRNA sequencing was used to analyze the gut microbiota composition and function in the control, SiO_2_, and DHQ groups. Overall, 1,196,988 usable reads and 5997 OTUs were obtained from 18 samples. The Sobs and Shannon diversity rarefaction curves approached a plateau, indicating that the sequencing data adequately represented the community (Figure 2A,B). In the SiO_2_ group, the microbiome's alpha diversity decreased compared to the control group, indicating a decrease in species variety. Treatment with DHQ significantly boosted Sobs, Shannon, Ace, and Chao indexes, as shown in Figure 2C–F. Principal component analysis (PCA) utilizing Bray–Curtis dissimilarity showed notable changes in the composition of gut microbiota between SiO_2_ and DHQ groups, with a closer proximity observed between control and DHQ groups compared to SiO_2_ and DHQ groups (Figure 2G). Similarly, the system clustering tree analysis showed that the DHQ group was closer to the control group than to the SiO_2_ group, consistent with the findings of the PCA analysis (Figure 2H).

**FIGURE 3:**
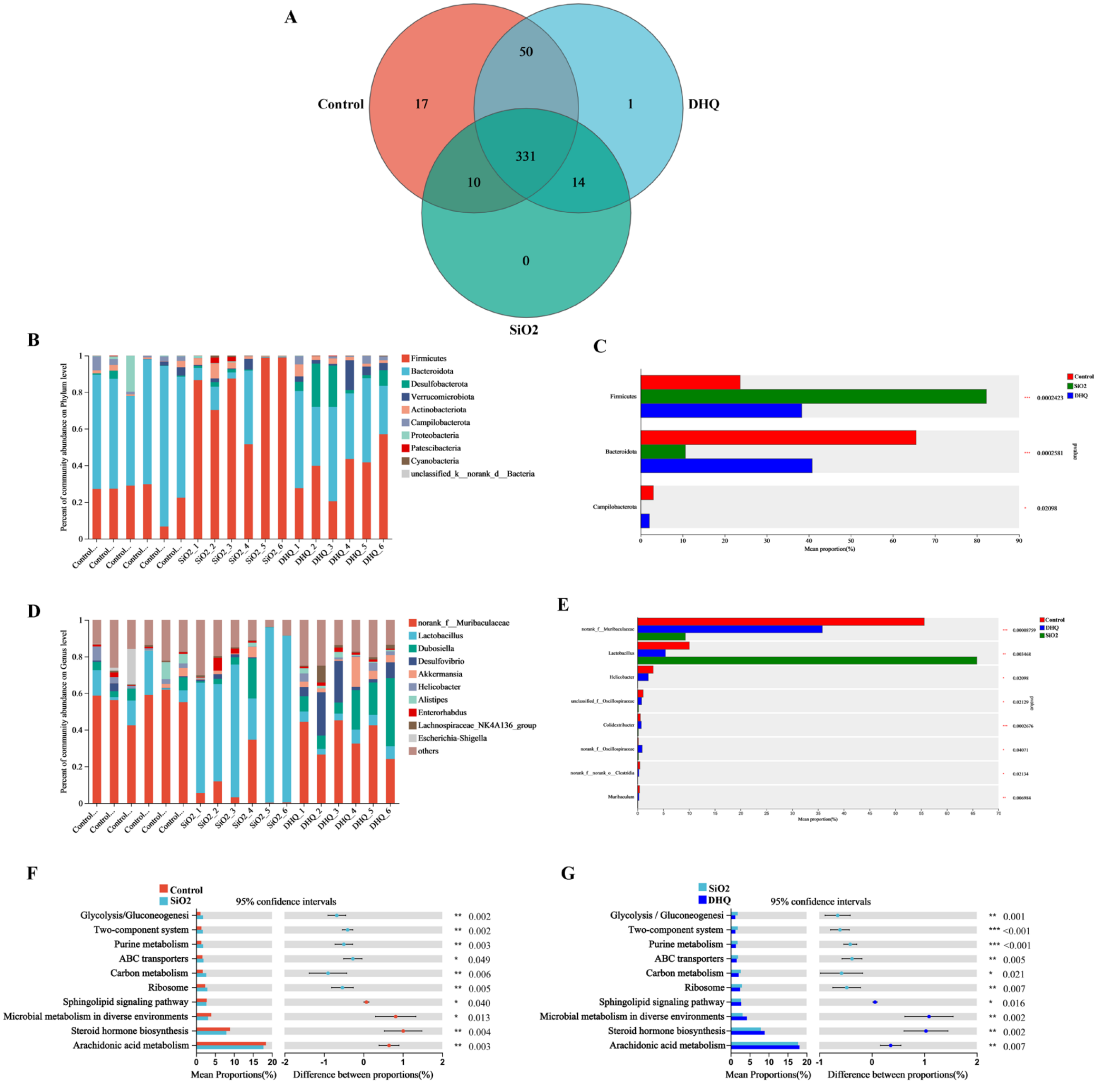
Analysis of microbial community differences between groups. (A) The different numbers of OTUs were visualized in a Venn diagram. (B, C) DHQ treatment changed bacteria at the phylum level in silicosis model mice. (D, E) DHQ treatment affected bacteria at the genus level in silicosis model mice. (F, G) The differential metabolic pathways of DHQ on silicosis model mice were predicted using PICRUSt analysis based on the 16S rRNA sequencing data. Data are presented as mean ± SD, *p < 0.05, **p < 0.01.

We conducted additional research on the variations in the proportions of different species of gut bacteria. The venn diagram showed that 331 OTUs were overlapped among three groups, while 341 OTUs were found in both the control and SiO_2_ groups, 381 in the control and DHQ groups, and 345 in the SiO_2_ and DHQ groups (Figure 3A). Compared to the control group, the SiO2 group showed a higher abundance of *Firmicutes* and a lower abundance of *Bacteroidota* at the phylum level. In silicosis model mice, the DHQ treatment reduced the *Firmicutes* to *Bacteroidetes* ratio (Figure 3B,C). At the genus level, *Lactobacillus* had significantly higher relative abundances, while *Muribaculaceae* had significantly lower relative abundances in the SiO_2_ group compared to the control group. The administration of DHQ resulted in a notable reduction in the relative abundance of *Lactobacillus* and a marked increase in the relative abundance of *Muribaculaceae* compared to the SiO_2_ (Figure 3D,E).

Furthermore, we used PICRUSt analysis to anticipate the potential pathways associated with the varying gut microbiota at the genus level. Figure 3F,G displays the top 10 metabolic pathway terms with the highest proportion and a *p* < 0.05 for the comparison among the groups. Differential metabolic pathways were identified as those that either rose in the SiO_2_ group while falling in the DHQ group, or the reverse. In the SiO_2_ group, there was a reduction in the levels of sphingolipid signaling pathway, microbial metabolism in various environments, steroid hormone biosynthesis, and arachidonic acid metabolism when contrasted with the control group. In contrast, the abundance of ribosomes, carbon metabolism, ABC transporters, purine metabolism, two‐component systems, and glycolysis/gluconeogenesis was greater in the SiO_2_ group than in the control group (Figure 3F). The DHQ group had higher levels of sphingolipid signaling pathway, microbial metabolism in various environments, steroid hormone biosynthesis, and arachidonic acid metabolism compared to the SiO_2_ group. Conversely, ABC transporters, the ribosome, carbon metabolism, purine metabolism, two‐component system, and glycolysis/gluconeogenesis were lower in the DHQ group (Figure 3G).

### 3.3 Effects of DHQ on Serum Metabolism in Silicosis Model Mice

**FIGURE 4:**
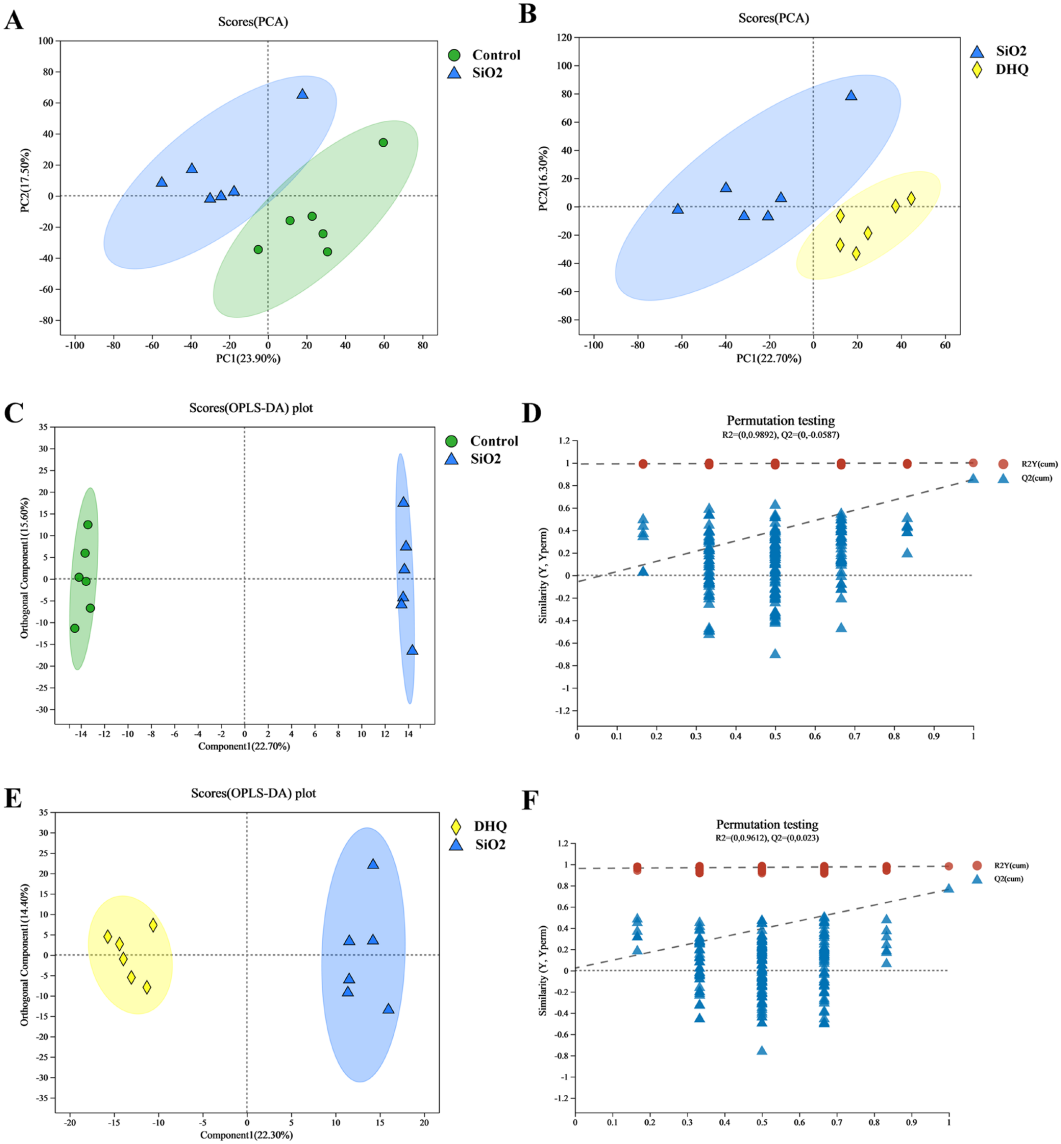
Scores plot of PCA and OPLS‐DA analysis from metabolic profiles of different serum samples. (A, B) PCA scores plots. (C–F) OPLS‐DA scores plots and permutation test of OPLS‐DA.

The Untargeted metabolomics was utilized to examine alterations in serum metabolites. The PCA model showed a distinct separation between the groups, as seen in Figure 4A,B. Hence, we utilized orthogonal partial least squares discriminant analysis (OPLS‐DA) to enhance the visualization of the metabolic changes observed among the different groups. The OPLS‐DA analysis revealed notable differences in metabolomic profiles among the three groups, as depicted in Figure 4C,E. Testing the model's reproducibility and data fit was recommended by avoiding the classification from supervised learning. The OPLS‐DA model underwent 200 response sorting tests to establish the stochastic model and determine the *R* ^2^ and *Q* ^2^ values. Comparing the control group with the SiO_2_ group, the OPLS‐DA model had *R* ^2^ and *Q* ^2^ values of 0.989 and −0.059, whereas the comparison between the DHQ group and the SiO_2_ group had *R* ^2^ and *Q* ^2^ values of 0.961 and 0.023, respectively. The findings suggested that the OPLS‐DA models were robust (Figure 4D,F).

**FIGURE 5:**
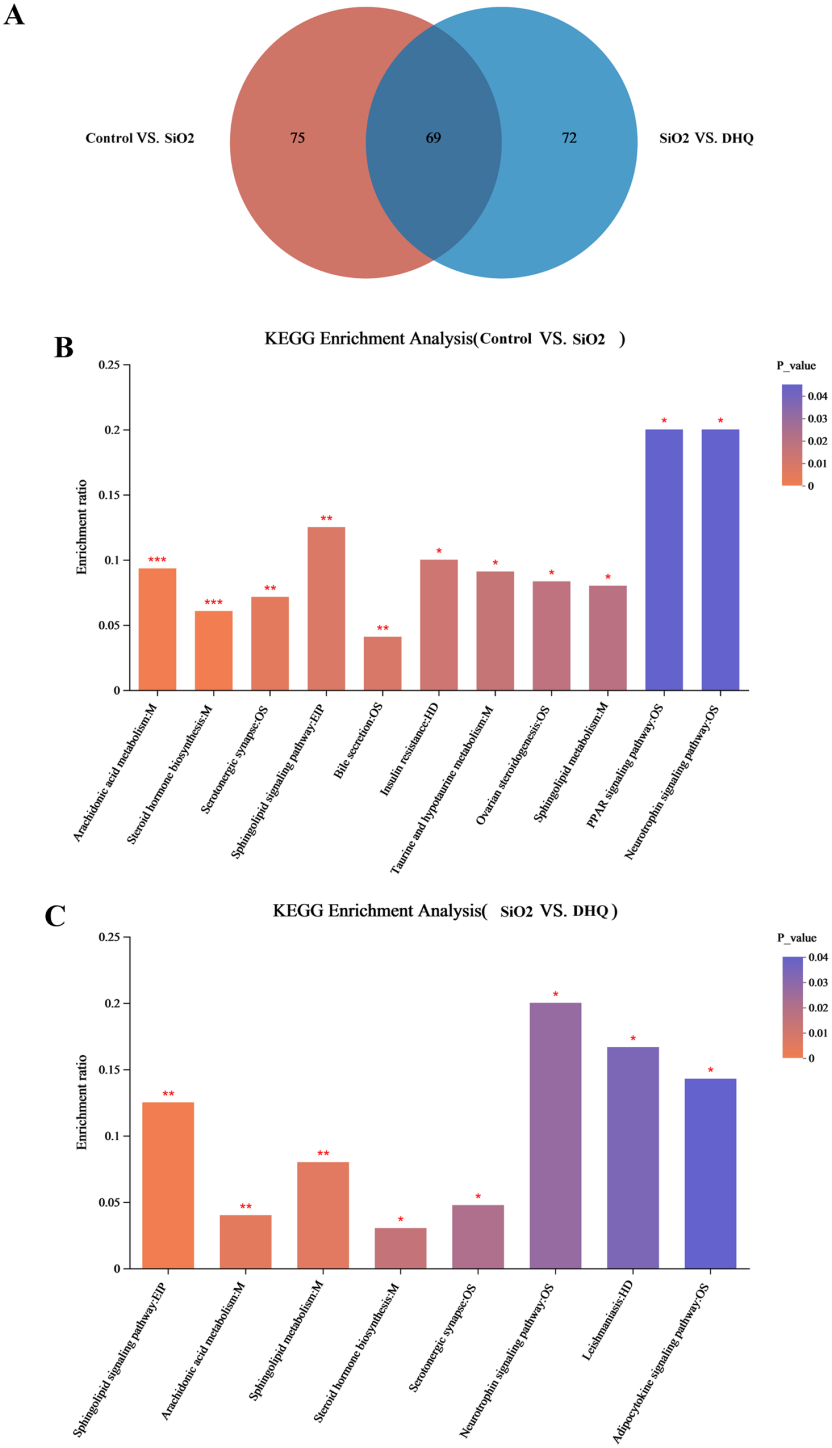
Pathway enrichment analysis of differential metabolites. (A) Venn diagram of differential metabolites. (B, C) KEGG enrichment analysis of differential metabolites. Data are presented as mean ± SD, *p < 0.05, **p < 0.01.

Differential metabolites were identified as those with a VIP score over 1 and a *p*‐value under 0.05 when comparing the groups. Figure 5A displayed the quantities of distinct metabolites among the control group, SiO_2_ group, and DCH group. Table 1 listed the top 25 differential metabolites terms between the groups with the highest enrichment and VIP. Furthermore, we sent the distinct metabolites to the KEGG platform to examine the associated pathways. Significant metabolic pathways involving arachidonic acid metabolism, steroid hormone production, serotonergic synapse, sphingolipid signaling pathway, bile secretion, insulin resistance, taurine and hypotaurine metabolism, ovarian steroidogenesis, sphingolipid metabolism, PPAR signaling pathway, and neurotrophin signaling pathway were found between the control and SiO_2_ groups (Figure 5B). Significant metabolic pathways including sphingolipid signaling pathway, arachidonic acid metabolism, steroid hormone biosynthesis, serotonergic synapse, neurotrophin signaling pathway, leishmaniasis, and adipocytokine signaling pathway were found between the SiO_2_ and DHQ groups (Figure 5C). The analysis of 16S rRNA sequencing and untargeted metabolomics revealed common pathways, including arachidonic acid metabolism, steroid hormone biosynthesis, and the sphingolipid signaling pathway.

**TABLE 1:** The differential metabolites in serum after DHQ treatment.

NO	Metabolites	Retention time	m/z	Formula	VIP		Trend		Pathway
					S vs. C	D vs. S	S vs. C	D vs. S	
1	(±) 7‐HDoHE	8.08	327.23	C_22_H_32_O_3_	1.11	1.54	↑*	↓**	—
2	Ent‐Prostaglandin E2	5.99	335.22	C_20_H_32_O_5_	1.93	1.61	↑***	↓**	—
3	PGB1	7.67	359.22	C_20_H_32_O_4_	1.60	1.47	↓**	↑*	—
4	11beta‐PGE2	5.75	351.21	C_20_H_32_O_5_	1.94	1.61	↑***	↓**	—
5	Hepoxilin B3	7.76	335.22	C_20_H_32_O_4_	1.44	1.55	↑*	↓**	Arachidonic acid metabolism
6	Arachidonic Acid	8.33	305.24	C_20_H_32_O_2_	1.2816	1.28	↑**	↓**	Arachidonic acid metabolism
7	Hydroxytyrosol 1‐O‐glucoside	4.46	297.10	C_14_H_20_O_8_	2.94	3.61	↓***	↑***	—
8	SM (d18:0/16:1(9Z))	11.33	747.56	C_39_H_79_N_2_O_6_P	1.00	1.02	↑**	↓**	Sphingolipid metabolism; Sphingolipid signaling pathway
9	3b,17b‐Dihydroxyetiocholane	7.52	337.23	C_19_H_32_O_2_	1.09	1.29	↓*	↑*	—
10	Nicotinic acid adenine dinucleotide	1.04	702.05	C_21_H_27_N_6_O_15_P_2_^+^	2.92	3.20	↓***	↑***	Metabolic pathways; Nicotinate and nicotinamide metabolism
11	11‐Hydroxyandrosterone	5.75	289.22	C_19_H_30_O_3_	2.62	1.98	↓***	↑**	—
12	15 (R)‐PGF2alpha	5.77	337.23	C_20_H_34_O_5_	1.85	1.43	↑***	↓**	—
13	15‐keto‐Prostaglandin E2	5.74	349.20	C_20_H_30_O_5_	1.39	1.11	↑***	↓*	—
14	8‐iso Prostaglandin A2	6.30	333.20	C_20_H_30_O_4_	1.69	1.24	↑**	↓*	—
15	16,16‐dimethyl‐PGD2	6.33	363.25	C_22_H_36_O_5_	1.34	1.20	↑*	↓*	—
16	8‐iso‐15‐keto‐PGE2	5.96	351.21	C_20_H_30_O_5_	1.5529	1.21	↑***	↓*	—
17	Prostaglandin E1	5.7768	319.22	C_20_H_34_O_5_	1.93	1.49	↓***	↑**	—
18	Cortolone	6.51	365.23	C_21_H_34_O_5_	1.62	1.19	↓**	↑*	Steroid hormone biosynthesis
19	14,15‐DiHETrE	8.46	321.24	C_20_H_34_O_4_	1.16	1.30	↑*	↓**	Arachidonic acid metabolism; Serotonergic synapse
20	13,14‐Dihydro PGE1	6.36	321.24	C_20_H_36_O_5_	1.27	1.18	↓*	↑*	—
21	5′‐CMP	1.00	324.05	C_9_H_14_N_3_O_8_P	1.59	1.64	↓***	↑**	—
22	Flurandrenolide	7.39	471.19	C_24_H_33_FO_6_	1.55	1.57	↓**	↑**	—
23	Vanilloloside	4.17	297.10	C_14_H_20_O_8_	3.10	3.47	↑***	↓***	—
24	21‐Deoxycortisol	5.30	347.22	C_21_H_30_O_4_	1.60	1.70	↓**	↑**	Steroid hormone biosynthesis
25	3,4‐Methylenesebacic acid	5.80	225.11	C_12_H_18_O_4_	2.09	2.26	↑*	↓*	—

*Note:* ↑, content increased; ↓, content decreased.

Abbreviations: C, control group; D, DHQ group; S, SiO_2_ group.

* *p* < 0.05.

** *p* < 0.01.

*** *p* < 0.001.

### 3.4 Correlation Analysis of Physiological Data, Serum Metabolites, and Gut Microbiota

**FIGURE 6:**
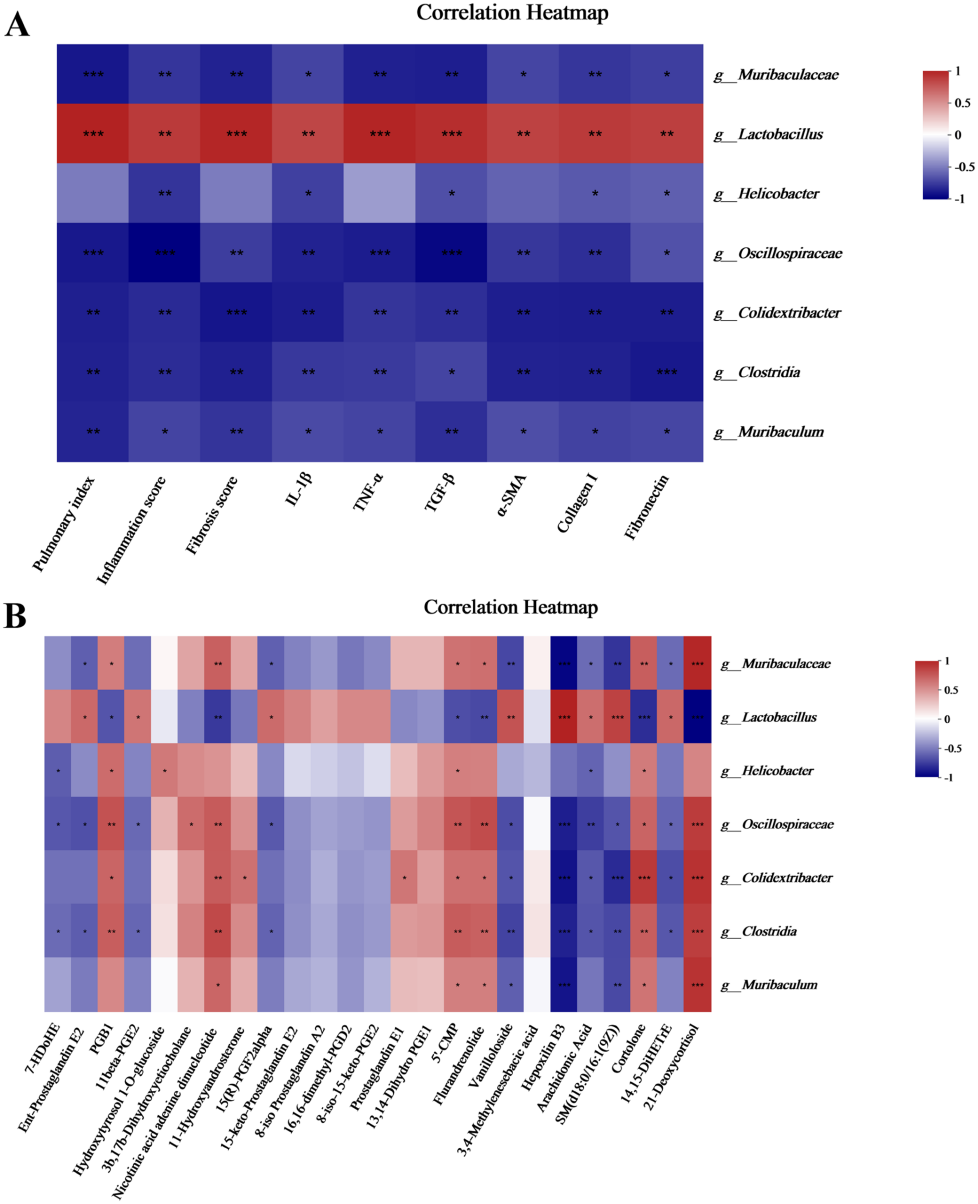
Spearman correlation analysis of physiological data, serum metabolites, and gut microbiota. (A) Heatmap of correlation between gut differential microbiota and physiological indices. (B) Heatmap of correlation between gut differential microbiota and serum differential metabolites. *p < 0.05, **p < 0.01, ***p < 0.001.

In Figure 6A, it was evident that *Lactobacillus* had shown significant positive correlations with the pathological changes in silicosis model mice, whereas *Muribaculaceae* were inversely related to the pathological changes observed in silicosis model mice. Additionally, *Muribaculaceae* showed negative correlations with arachidonic acid metabolites and positive correlations with the cortolone and 21‐Deoxycortisol. *Lactobacillus* showed positive associations with the majority of arachidonic acid metabolites and negative associations with cortolone and 21‐Deoxycortisol (Figure 6B).

## 4 Discussion

This research explored the mechanisms that contribute to the healing properties of DHQ on silica‐induced pulmonary fibrosis in a mice model, utilizing 16S rRNA sequencing of gut bacteria and serum metabolomics. Consistent with our previous study (Yuan et al. 2022), the present study also showed that DHQ protected against lung fibrosis caused by silica (Figure 1). DHQ is a commonly utilized compound with antifibrotic and antioxidant properties. The study by Guo et al. revealed that DHQ can attenuate ventricular fibrosis following pressure overload (Guo et al. 2015). Moreover, Ding et al. demonstrated that DHQ exhibited protective effects on the kidneys, particularly by decreasing renal histopathological damage in diabetic nephropathy through the inhibition of reactive oxygen species (ROS) and NOD‐like receptor thermal protein domain associated protein 3 (NLRP3) inflammasome activation (Ding et al. 2018). Additionally, research by Liu et al. revealed that DHQ decreased inflammation and alleviated liver fibrosis triggered by CCl4 through the regulation of the PI3K/AKT/mTOR and TGF‐β1/Smads pathways (X. Liu et al. 2021). Moreover, Impellizzeri et al. reported that DHQ ameliorated pulmonary fibrosis triggered by bleomycin (Impellizzeri et al. 2015). Furthermore, Unver et al. reported that DHQ shielded the pulmonary tissue against the harmful impacts of cisplatin (Unver et al. 2019). The research conducted previously has confirmed the anti‐inflammatory and anti‐fibrotic attributes of DHQ. In our findings, it was evident that administering DHQ led to a notable decrease in collagen accumulation caused by silica in C57/BL6 mice when compared to the SiO_2_ group. Furthermore, our findings indicated a reduction in IL‐1β, TNF‐α, and TGF‐β levels after administering DHQ. These results indicated that DHQ could alleviate fibrosis caused by SiO_2_ by inhibiting inflammation.

To identify alterations in the microbiological composition linked to silica‐induced pulmonary fibrosis, we examined the gut microbiome changes in silicosis model mice following DHQ treatment. The SiO_2_ group had less alpha diversity in their gut microbiota than the control group, but alpha diversity increased following DHQ treatment in our findings. Our results aligned with other researchers' studies indicating that patients with silicosis exhibited a decreased alpha diversity in their gut microbiota community (Zhou et al. 2019). Analysis using PCA and a clustering tree system showed that the beta diversity of gut microbiota was impacted by DHQ treatment. Interestingly, the beta diversities of rats treated with DHQ were more similar to those in the control group than to those in the SiO_2_ group. The ratio between *Firmicutes* to *Bacteroidetes* is closely related to idiopathic pulmonary fibrosis (Garzoni et al. 2013). Patients diagnosed with idiopathic pulmonary fibrosis exhibited a notable rise in the abundance of *Firmicutes* and a substantial decline in *Bacteroidetes* compared to healthy individuals (Garzoni et al. 2013; Invernizzi et al. 2021). Our findings supported the previous observations by showing a significant rise in the ratio of *Firmicutes* to *Bacteroidetes* in the SiO_2_ group, which was subsequently reduced following DHQ treatment. Our research found that the SiO_2_ group had an increased abundance of *Lactobacillus* at the genus level. Studies have linked *Lactobacillus* to idiopathic pulmonary fibrosis. A study found that there was a higher presence of *Lactobacillus* in the lung tissues of patients with IPF compared to the control group (Invernizzi et al. 2021). Moreover, Harata et al. found that intranasal *Lactobacillus rhamnosus* significantly increased mRNA expression of IL‐1, TNF, and monocyte chemotactic protein 1 (MCP‐1) (Harata et al. 2010). Given that proinflammatory cytokines and chemokines are involved in the development of IPF, *Lactobacillus* might contribute to its pathophysiology (Mutsaers et al. 2023). *Lactobacillus* is capable of generating lactic acid, which can trigger the transformation of fibroblasts into myofibroblasts through the activation of TGF‐β (Uribe et al. 2018). Hence, it is also possible that lactic acid produced by *Lactobacillus* contributes to the progression of IPF. Meanwhile, the relative abundances of *Muribaculaceae* were increased post DHQ treatment. Feng et al. discovered that *Muribaculaceae* were negatively correlated with inflammation and oxidative stress, and panaxanediol may have a therapeutic effect on NAFLD by increasing the abundance of *Muribaculaceae* (Feng et al. 2023). A study had shown a negative correlation between the levels of *Muribaculaceae* and blood lipids (Yang et al. 2024). A study indicated that Pu‐erh tea enhanced weight loss by increasing the levels of beneficial bacteria like *Muribaculaceae* (Ye et al. 2021). Phytic acid was found to improve hepatic steatosis, inflammation, and oxidative stress by boosting the presence of probiotic bacteria *Muribaculaceae* (Ran et al. 2022). Considering these findings, DHQ might improve silica‐induced pulmonary fibrosis by affecting the mouse gut microbiota. Subsequent correlation analysis indicated that *Lactobacillus* was significantly positively associated with physiological data, including serum IL‐1β, TNF‐α, TGF‐β, and indexes related to pulmonary fibrosis, and *Muribaculaceae* was obviously negatively associated with physiological data. Therefore, *Lactobacillus* and *Muribaculaceae* might be the targets of DHQ to attenuate inflammation and pulmonary fibrosis. PICRUSt analysis was used to predict metabolic pathways associated with changes in the gut microbiota. DHQ treatment mainly reversed the disorders of metabolic pathways in arachidonic acid metabolism, steroid hormone biosynthesis, and sphingolipid signaling pathway in silicosis model mice.

In order to better illustrate the changes in the metabolism, we further investigated the serum metabolites levels through untargeted metabolomic. Serum untargeted metabolomics analysis using OPLS‐DA revealed distinct metabolic profiles among the control, SiO_2_, and DHQ groups, suggesting that DHQ has an impact on metabolic profiles in a silica‐induced silicosis mouse model. DHQ‐induced changes in metabolites also triggered the alterations in multiple KEGG pathways. Indeed, DHQ significantly decreased lipid metabolites and then weakened the production of arachidonic acid and its metabolites. Arachidonic acid is known to be a precursor of several potent proinflammatory mediators and now has become the target of anti‐inflammatory research (ElKhatib et al. 2023; Pereira et al. 2024). A study indicated a positive correlation between the expression of α‐SMA, collagen, and fibronectin in fibroblasts and the presence of 15‐hydroxyeicosatetraenoic acid (15‐HETE). Furthermore, exogenous 15‐HETE can induce phenotypic changes in the extracellular matrix of fibroblasts via the TGF‐β1/SMad2/3 signaling pathway (Zhang et al. 2014). Here, the dramatic decrease of arachidonic acid and its metabolites in serum induced by DHQ might be the potential therapeutic targets for systemic inflammation. In the current study, the supplementation of DHQ weakened the production of sphingomyelin by affecting sphingolipid signaling pathways. Sphingomyelin plays a crucial role in plasma membrane composition and controls various cellular responses such as vascular permeability, cell survival, cell activation, and airway smooth muscle functions (Uhlig and Gulbins 2008). A recent study highlighted the role of sphingomyelin metabolism in conditions such as cystic fibrosis, asthma, respiratory tract infections, and acute lung injuries (Uhlig and Gulbins 2008). Furthermore, we noted that the metabolites of cortisol and steroid hormone biosynthesis pathway were activated by DHQ. Several studies had confirmed the important roles of steroid hormone biosynthesis pathway, which can alleviate inflammation (Payne and Freishtat 2012; Schmidt et al. 2006; Zhou et al. 2023). Payne reported that steroid hormone can reduce inflammatory responses in asthma through common nuclear factor κB‐mediated downstream events. Some studies had also reported that steroid hormone metabolites were closely related to the alleviation of rheumatoid arthritis (Schmidt et al. 2006; Zhou et al. 2023). A recent study indicated that a dose‐specific effect of CAPE treatment on circulating BA content, ileal BSH‐FXR signaling and gut microbiota, which can improve glucose and lipid dyshomeostasis through reducing inflammation (Cai et al. 2025). DHQ might improve metabolic disorders caused by SiO_2_ through increasing steroid hormone metabolism. Overall, the anti‐inflammatory and antifibrotic properties of DHQ were attributed to metabolic changes. Combined with the results of PICRUSt analysis and untargeted metabolomics analysis, the study identified three key metabolic pathways including arachidonic acid metabolism, steroid hormone biosynthesis, and sphingolipid signaling pathways. Our further correlation analysis indicated that *Lactobacillus* was significantly positively correlated with SM, arachidonic acid and its metabolites, and significantly negatively correlated with cortolone and 21‐deoxycortisol, while *Muribaculaceae* was significantly negatively associated with SM, arachidonic acid and its metabolites, and significantly positively correlated with cortolone and 21‐deoxycortisol. Therefore, we theorized that DHQ may regulate the imbalances in arachidonic acid metabolism and steroid hormone biosynthesis and sphingolipid signaling pathways by improving the gut microbiome.

Despite the aforementioned findings, our study is subject to certain limitations. Firstly, there are significant differences between the gut microbiota composition and serum metabolites of mice and those of humans. Consequently, the alterations in gut microbiota and serum metabolites observed following DHQ administration in this study may not accurately represent the human response. Secondly, we did not conduct an extensive investigation into the complex molecular mechanisms underlying the interaction between gut microbiota and serum metabolites in the context of silica‐induced pulmonary fibrosis. Nevertheless, our research provides evidence that DHQ can mitigate silica‐induced pulmonary fibrosis, with the interplay between gut microbiota and serum metabolites following DHQ treatment playing a crucial role in the amelioration of silicosis. Targeting gut microbiota and serum metabolites may present a promising avenue for the development of novel therapeutic interventions for silicosis.

## 5 Conclusion

**FIGURE 7:**
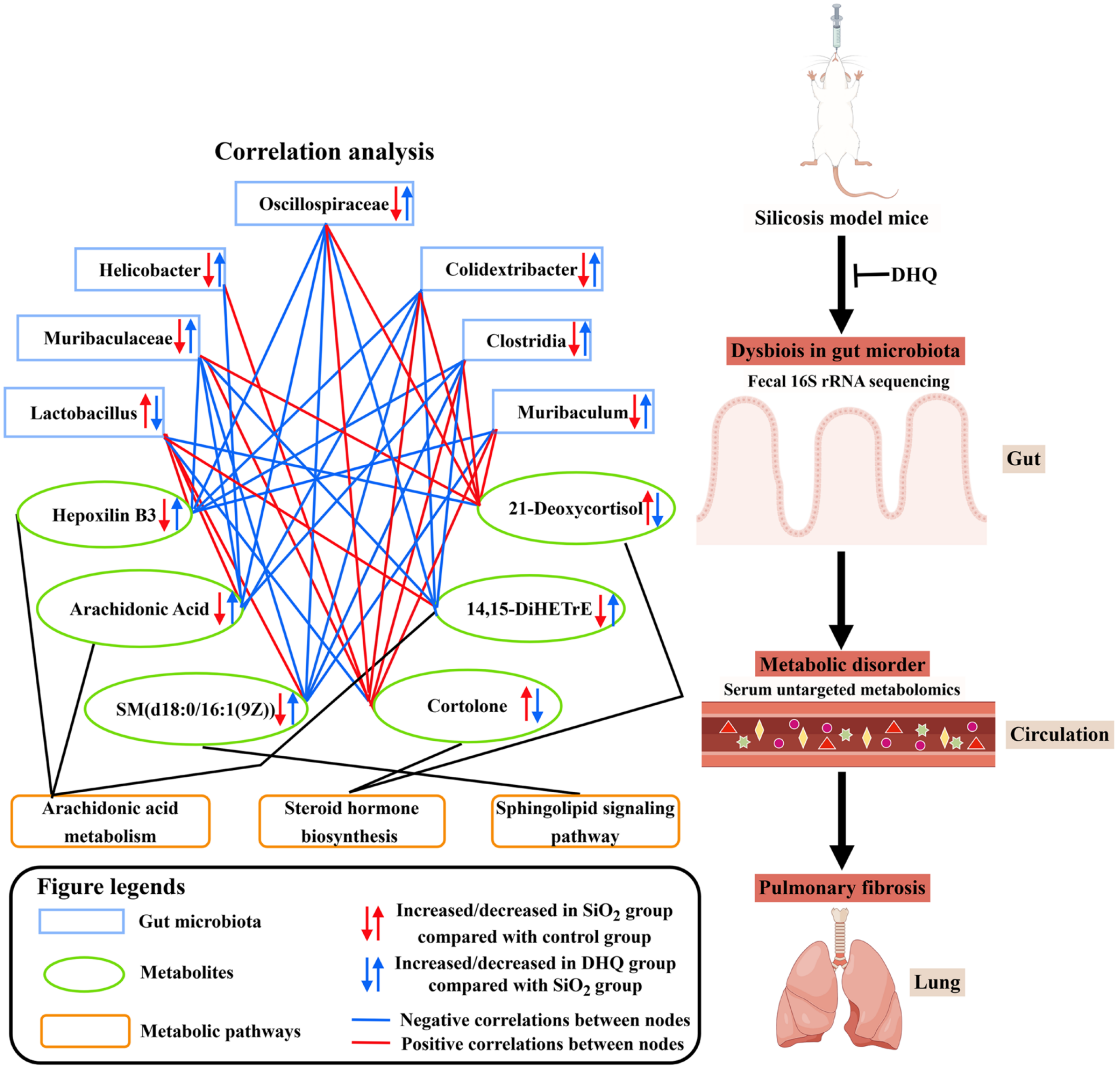
Schematic diagram of potential mechanisms of protective effect against silica‐induced pulmonary fibrosis in C57BL/6 mice.

By integrating multiomic analyses, we uncovered that DHQ treatment attenuates inflammation and silica‐induced pulmonary fibrosis potentially by modulating the serum metabolome and gut microbiota. Further analysis indicated that the beneficial effects of DHQ were primarily attributed to the alteration of gut microbiota (particularly *Muribaculaceae* and *Lactobacillus*) mediated by serum metabolites (Figure 7). These findings will help identify the effective therapeutic direction to alleviate silica‐induced pulmonary fibrosis.

## Author Contributions

**Zunqiong Ke:** project administration (equal), resources (equal), writing – original draft (equal). **Lishu Lin:** project administration (equal), writing – original draft (equal). **Yunhong Long:** data curation (equal), formal analysis (equal), methodology (equal), project administration (equal), resources (equal), software (equal), visualization (equal). **Wenhui Zhang:** data curation (equal), formal analysis (equal), methodology (equal), project administration (equal), software (equal), visualization (equal). **Jianmin Guo:** conceptualization (equal), methodology (equal), resources (equal). **Leyong Yuan:** conceptualization (equal), methodology (equal), resources (equal).

## Funding

This work was supported by Guangdong Zhong Nanshan Medical Foundation Research Funding Project, ZNSXS‐20240119; Medical Scientific Research Foundation of Guangdong Province, B2025556; Shenzhen Science and Technology Innovation Commission, JCYJ20220530112608018; Futian Healthcare Research Project, FTWS019.

## Conflicts of Interest

The authors declare no conflicts of interest.

## Data Availability

The data that support the findings of this study are available from the corresponding author upon reasonable request.
